# Supplementary material for: Insular cortex predictions regulate glucose homeostasis
Source: bioRxiv. 2025 Dec 11:2025.12.09.693191. Preprint. [Version 1] doi: 10.64898/2025.12.09.693191 (PMC12822591; doi:10.64898/2025.12.09.693191)
Supplement: Supplementary file 1 [file NIHPP2025.12.09.693191v1-supplement-3.pdf]

bioRxiv preprint doi: https://doi.org/10.64898/2025.12.09.693191; this version posted December 11, 2025. The copyright holder for this preprint (which was not certified by peer review) is the author/funder, who has granted bioRxiv a license to display the preprint in perpetuity. It is made available under a CC-BY 4.0 International license.

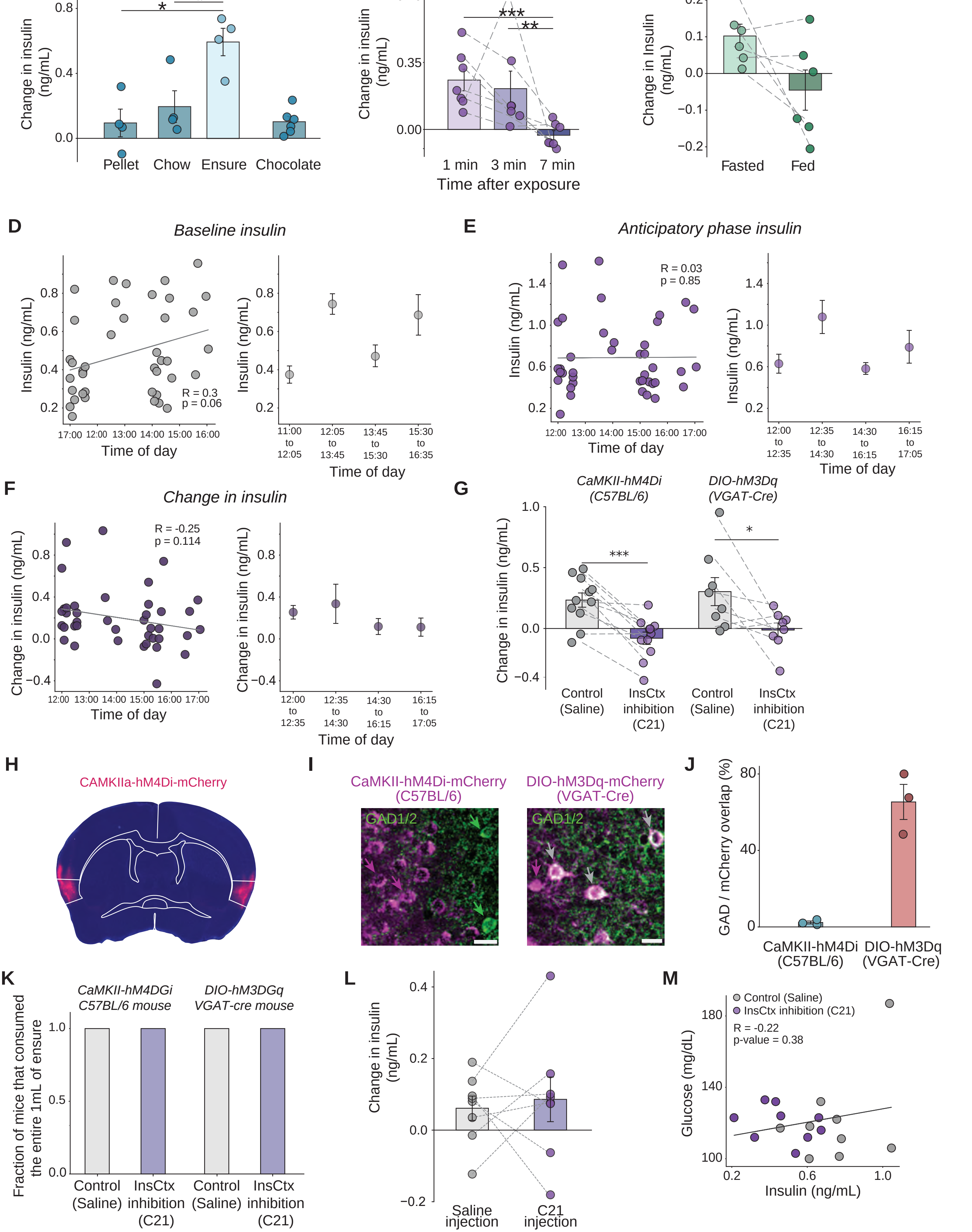

bioRxiv preprint doi: https://doi.org/10.64898/2025.12.09.693191; this version posted December 11, 2025. The copyright holder for this preprint (which was not certified by peer review) is the author/funder, who has granted bioRxiv a license to display the preprint in perpetuity. It is made available under a CC-BY 4.0 International license.

Supp. Fig. 1: Additional experiments and analyses for the learned anticipatory insulin release assay and for chemogenetic inhibition experiments.

(A) Comparison of anticipatory insulin release across 4 different types of food. Ensure was the food type that elicited the highest and most consistent anticipatory insulin release ($n_{Pellet,\ Chow,\ Ensure}$=4, $n_{Chocolate}$=6, $p_{Ensurevs.Pellet}$=0.002, $p_{Ensurevs.Chow}$=0.01, $p_{Ensurevs.Chocolate}$=0.0009).

(B) Anticipatory insulin kinetics. Normalized change in insulin levels 1, 3 or 7 minutes after presenting the bowl of Ensure during the protocol, mice had similar levels in the first 1-3 minutes (n=7, p=0.68), declining thereafter to baseline after no further exposure (p_{1vs7}=0.002. P_{3vs7}=0.04).

(C) Overnight fasted mice (14 -18 hrs) had higher anticipatory insulin release compared to the same mice without fasting (n=6, p = 0.05).

(D-F) Analysis of the effect of time-of-day on insulin levels.

(D) No significant correlation between time-of-day and circulating baseline insulin levels in fasted mice, despite a positive trend (n=40, R= 0.3, p=0.06).

(E) No significant correlation between time-of-day and anticipatory insulin release levels (n=40, R= 0.03, p=0.85).

(F) No significant correlation between time-of-day and the change in insulin (n=40, R= 0-0.25, p=0.11).

(G) Re-analysis of the two different approaches to chemogenically inhibit InsCtx, without removing non-responders, show similar significant effect in anticipatory insulin (see Methods; CaMKII-hM4Di: n=11, p=0.0001; VGAT-hM3Dq: n=8, p=0.02).

(H) Example of coronal slice at bregma with bilateral hM4Di-mCherry expression. Scale bar: 1 mm.

(I) Example images of brains from slices at bregma showing specificity of viral expression. *Left*: AAV-CaMKII-hM4Di in C57BL/6 mice show very little overlap with anti-GAD1/2 immunostaining. *Right:* AAV-DIO-hM3Dq in a VGAT-Cre mic with relatively high overlap between virus expression and immunostaining. Magenta arrow: viral expression, green arrow: somatic anti-GAD1/2 labeling, grey arrows: neurons with overlap. Scale bar: 20 µm.

(J) Quantification of the percentage of overlap between neurons expressing the virus and labeled with the anti-GAD1/2 antibody for both cohorts (green and red out of all red). n_{CaMKII}= 104±22, n_{VGAT}= 114±5 neurons from 3 mice each.

(K) All mice, from both cohorts, consumed the total amount of Ensure given (1 mL) in both experimental days (n_{CaMKII}=9, n_{VGAT}=7).

(L) Control experiment for chemogenetic inhibition showing that C21 injection in mice expressing a non-chemogenetic virus in the InsCtx did not reduce anticipatory insulin release (n=8, 0.73).

(M) No significant correlation between glucose levels and insulin levels during the anticipatory phase (n=9, R = 0.081, p = 0.74), indicating that the increase in anticipatory insulin release in the control day is not triggered by increases blood glucose).

Data are presented as individual measurements (points) and mean (bars) ± SEM. Gray dashed lines represent the same mouse. P values were calculated by Student's paired two sample t-test (for **C,G,L**), Pearson correlation (for m), Spearman's rank test (for **D-F**), ANOVA with post-hoc Tukey test (for **A**), or repeated measures ANOVA with pairwise post-hoc test Benjamini-Hochberg (for **B**). * p<0.05, ** p<0.01.

bioRxiv preprint doi: https://doi.org/10.64898/2025.12.09.693191; this version posted December 11, 2025. The copyright holder for this preprint (which was not certified by peer review) is the author/funder, who has granted bioRxiv a license to display the preprint in perpetuity. It is made available under a CC-BY 4.0 International license.

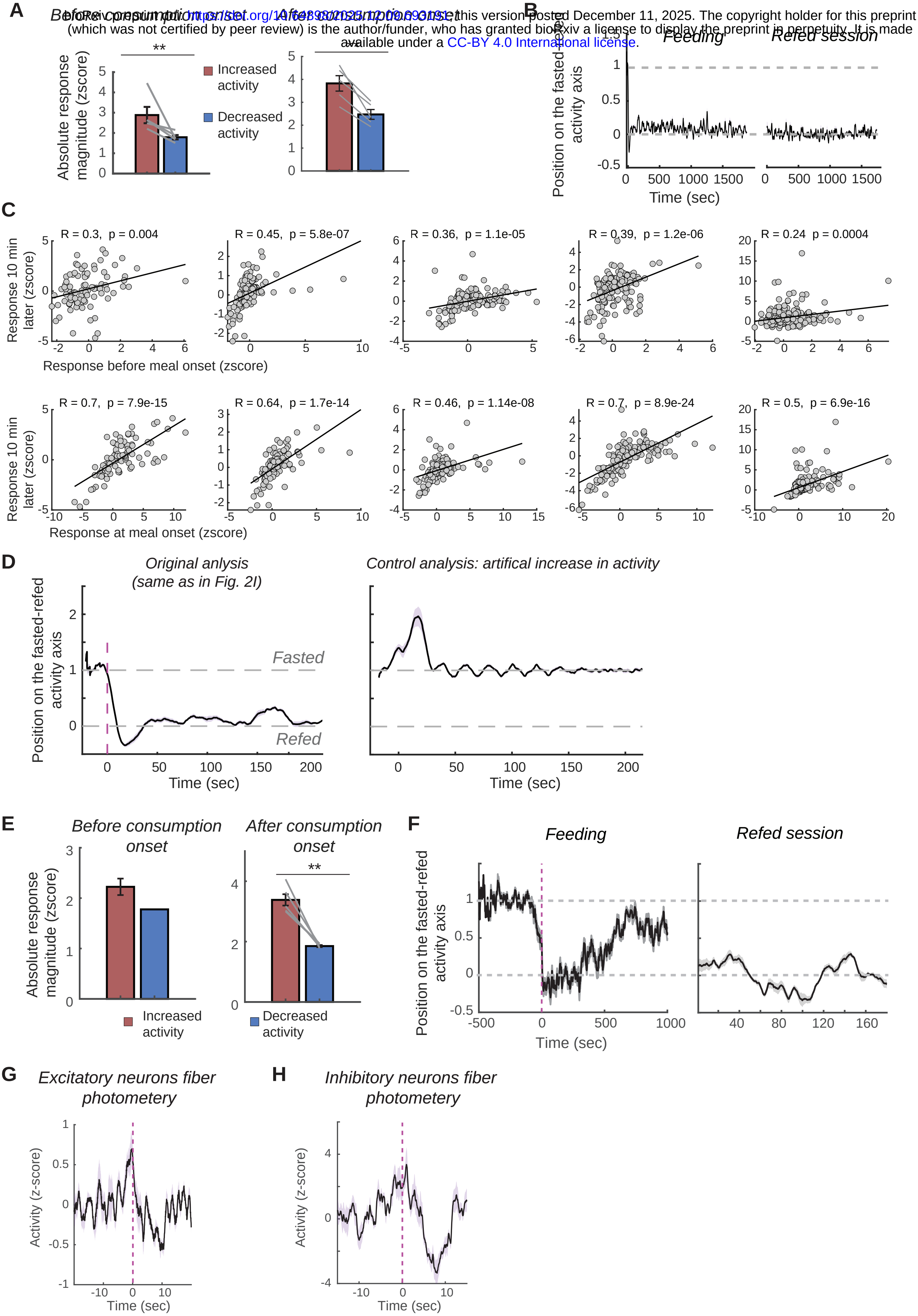

bioRxiv preprint doi: https://doi.org/10.64898/2025.12.09.693191; this version posted December 11, 2025. The copyright holder for this preprint (which was not certified by peer review) is the author/funder, who has granted bioRxiv a license to display the preprint in perpetuity. It is made available under a CC-BY 4.0 International license.

Supp. Fig. 2: Additional analyses for InsCtx two-photon imaging and one-photon miniscope imaging experiments.

(A) Absolute response magnitude of increased vs. decreased responses before (p = 0.03) and at (p = 0.002) meal onset.

(B) Position on the fasted-refed axis of population activity shown for the entire 30 min feeding session and the entire 30 min refed (post-meal) session. Note that activity remains stable around values of 0 in the refed session.

(C) Correlation across neurons per mouse between the activity before onset (top) or at meal onset (bottom) and the activity 10 min later. Note significant and high correlations but with substantially lower values 10 min later. Each plot column is from a different mouse.

(D) Control analysis for the specificity of InsCtx meal onset activity patterns. We artificially increased activity by multiplying the baseline activity level of each neuron by a factor of 2. This resulted in an opposite effect, suggesting the burst of activity at meal onset is a specific pattern, distinct from the baseline (fasted) pattern, regardless of overall activity levels.

(E) Same as 'A' but for miniscope data.

(F) Same as 'B' but for miniscope data.

(G-H) Bulk activity fiber phototmetry from excitatory neurons (**G**) and inhibitory (**H**). Vertical dashed line: licking onset. n= 4 mice for each group. Values are Mean ± SEM across mice. The p-values were calculeted by Student's paired t-test. * p<0.05, ** p<0.01

bioRxiv preprint doi: https://doi.org/10.64898/2025.12.09.693191; this version posted December 11, 2025. The copyright holder for this preprint (which was not certified by peer review) is the author/funder, who has granted bioRxiv a license to display the preprint in perpetuity. It is made available under a CC-BY 4.0 International license.

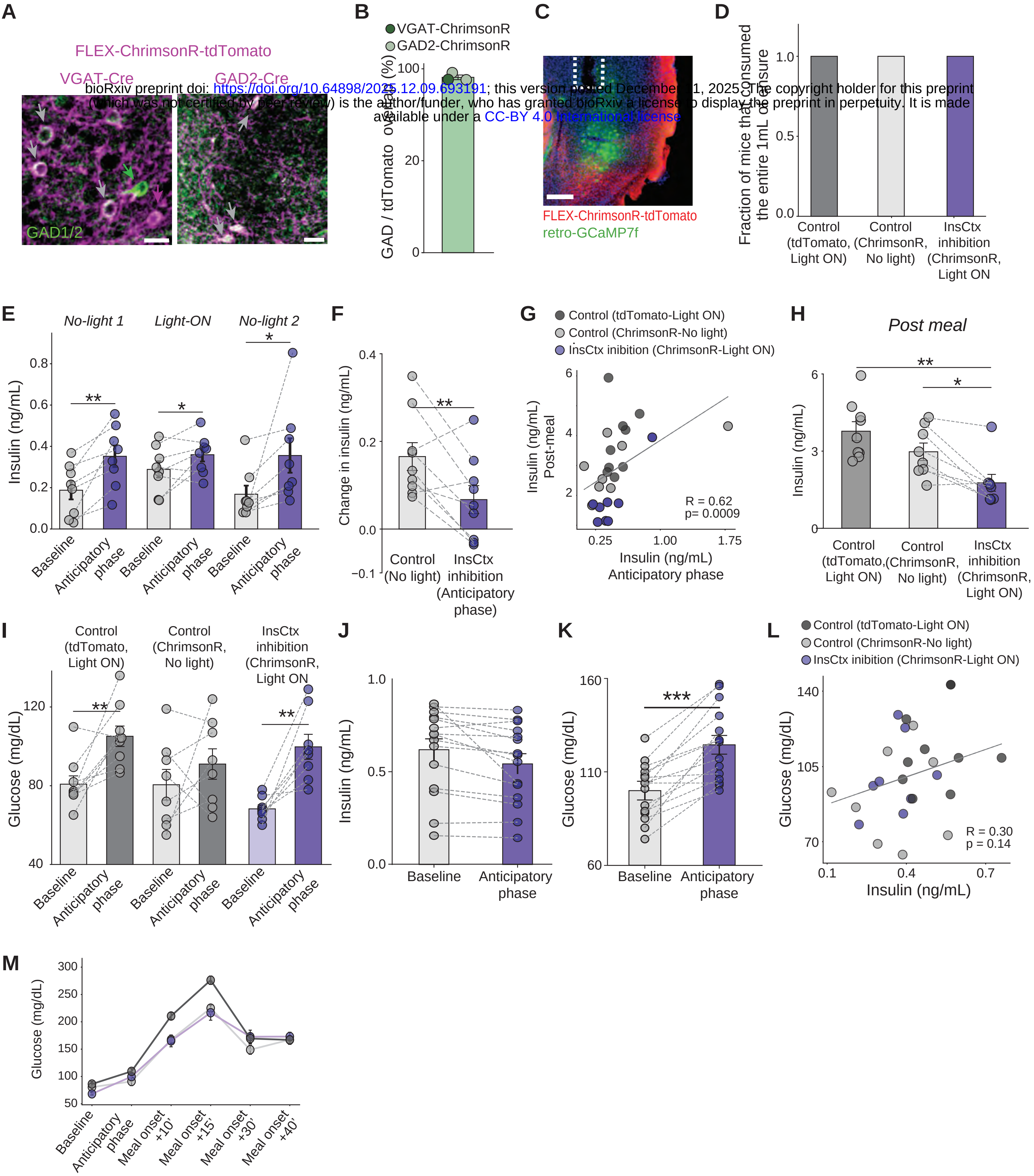

bioRxiv preprint doi: https://doi.org/10.64898/2025.12.09.693191; this version posted December 11, 2025. The copyright holder for this preprint (which was not certified by peer review) is the author/funder, who has granted bioRxiv a license to display the preprint in perpetuity. It is made available under a CC-BY 4.0 International license.

Supp. Fig. 3: Further control experiments and analyses for within-subject optogenetic inhibition experiments.

(A) Example images from brains slices at bregma showing specificity of viral expression. *Left*: AAV-FLEX-ChrimsonR in a VGAT-Cre mice shows high overlap with anti-GAD1/2 immunostaining. *Right:* AAV-FLEX-ChrimsonR in a GAD2-Cre mice with high percentage of overlap between virus expression and the antibody. Green arrow: anti-GAD1/2, grey arrows: neurons with overlap. Scale: 20 μm.

(B) Quantification of the percentage of overlap between neurons expressing the AAV and labeled with the anti-GAD1/2 antibody for both Cre lines shows high specificity of viral expression in interneurons. *Dark green*: VGAT-Cre, *light green*: GAD2-Cre n= 83 ±30 (mean ±SEM) neurons from 3 mice.

(C) Example section with co-expression of GCaMP7f (in excitatory projection neurons) and ChrimsonR (in inhibitory interneurons), and fiber placement. Scale bar: 200 μm.

(D) All mice consumed the total amount of Ensure given (1 mL) in all experimental days ($n_{tdTomato}$=9, $n_{ChrimsonR}$=8).

(E) Control for the order experimental condition (Light ON or OFF) demonstrates that the order of conditions does not underlie the effects of optogenetic inhibition as mice show normal anticipatory insulin the following week with no light during the protocol. First week: mice had no light during the protocol (No Light 1) and normal anticipatory insulin release (n=8, p=0.004). Second week: we turned on LED during the anticipatory phase (Light ON) and mice had reduced anticipatory insulin release (p=0.044, see Fig. 3). Third week: no light during the anticipatory phase (No Light 2) and mice recovered their anticipatory insulin release (p=0.006). See Fig. 3 for insulin change values per condition.

(F) Re-analysis of the effect of optogenetic inhibition of InsCtx on anticipatory insulin, without removing non-responders, demonstrates a similar effect (n=9, p=0.008).

(G) Re-analysis of the correlation between anticipatory and post-meal insulin, without removing non-responders, shows similar results (n=17, R=0.62, p=0.0009).

(H) Re-analysis of the effect of InsCtx optogenetic inhibition on post-meal insulin levels, without removing non-responders, shows a similar result ($n_{tdTomato}$=9, $n_{ChrimsonR}$=8, $p_{'Light\ control'}$=0.012, $p_{'opsin\ control'}$=0.002).

(I) Blood glucose levels at baseline and during the anticipatory phase, on the day of control illumination (tdtomato, Light ON), no light inhibition (ChrimsonR, control) and on the day with light on during the anticipatory phase (InsCtx inhibition, ChrimsonR, Light ON). There was a significant increase in blood glucose during the 'light ON' control day (n=9, p=0.008) and during the InsCtx inhibition day (n= 8, p = 0.002). Note that this variability in increased blood glucose in different days is likely due to the prolonged waiting period in our optogenetic experiments and cannot explain the decrease in anticipatory insulin upon optogenetic inhibition of InsCtx (see 'L' and Supp. Fig. 4B,C).

(J-K) Mice with no anticipatory phase insulin release during control experiments of the optogenetic protocols (**J**) still had significant increase in blood glucose (**K**, n=15, p=0.0003), likely related to stress-induced hyperglycemia in optogenetic experiments.

(L) No correlation between blood glucose levels and insulin levels during the anticipatory phase ($n_{tdTomato}$=9, $n_{ChrimsonR}$=8, R = 0.3, p = 0.14), indicating that the increase in blood glucose levels in the optogenetic inhibition day is not the reason for the lack of anticipatory insulin release. As mentioned in '**I**', there should have been a negative correlation in that case.

bioRxiv preprint doi: https://doi.org/10.64898/2025.12.09.693191; this version posted December 11, 2025. The copyright holder for this preprint (which was not certified by peer review) is the author/funder, who has granted bioRxiv a license to display the preprint in perpetuity. It is made available under a CC-BY 4.0 International license.

(M) Whole blood glucose curve. Glucose levels at 30 minutes post-meal onset show significantly less clearance (n=8, p = 0.045), suggesting glucose homeostasis impairment.

Data are presented as individual measurements (points) and mean (vertical bars) ± SEM. Gray dashed lines represent the same mouse. P values were calculated by Student's paired two sample t-test (for **E,F,G,I,J,K**), independent Student's t-test (for **H**), Wilcoxon (for **E,H,I**), Mann Whitney's U test (for **H**), Benjamini-Hochberg correction (**H**), Spearman's rank test (for **G,L**). *p<0.05, **p <0.01, ***p<0.001, *****p <0.00001.

bioRxiv preprint doi: https://doi.org/10.64898/2025.12.09.693191; this version posted December 11, 2025. The copyright holder for this preprint (which was not certified by peer review) is the author/funder, who has granted bioRxiv a license to display the preprint in perpetuity. It is made available under aCC-BY 4.0 International license.

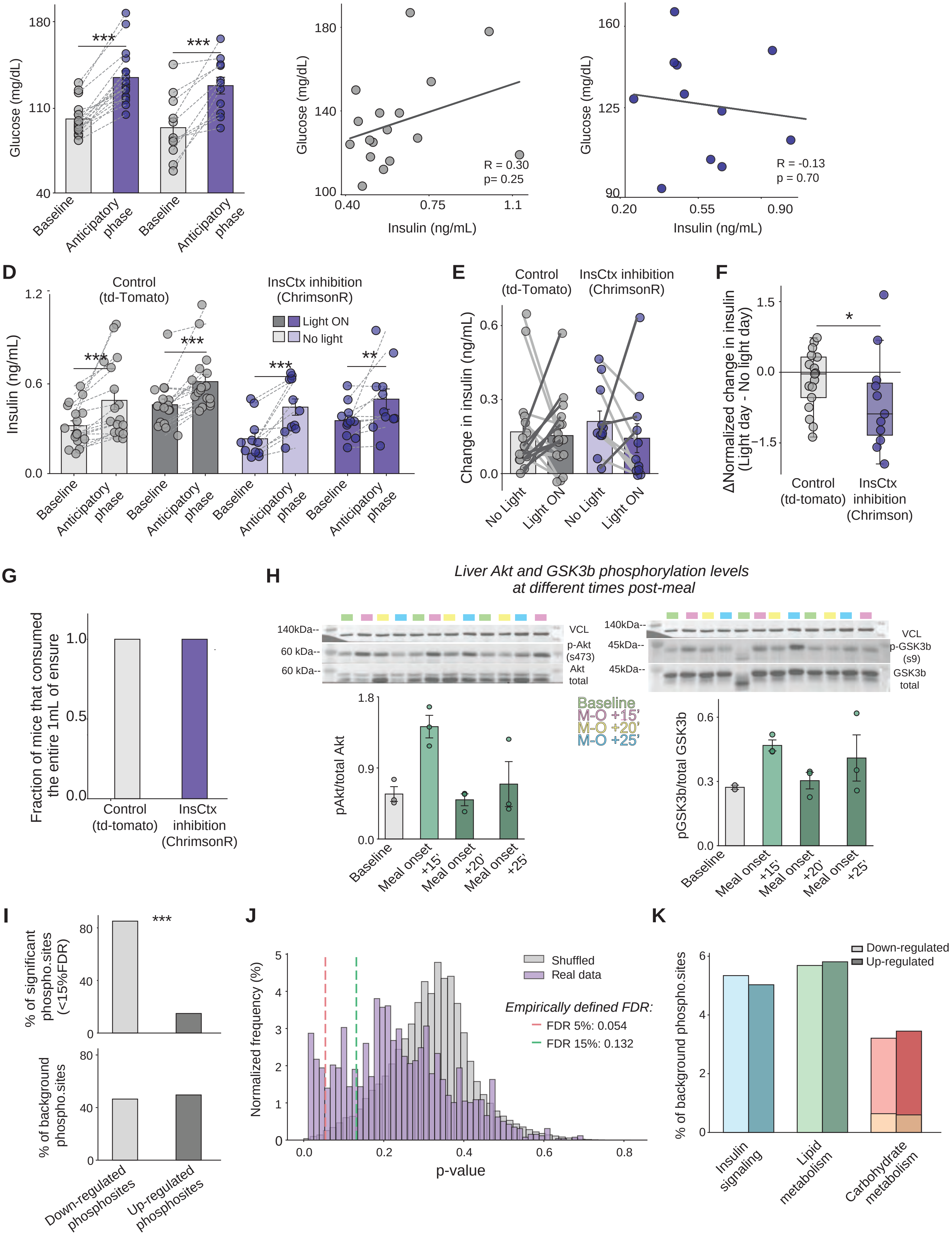

bioRxiv preprint doi: https://doi.org/10.64898/2025.12.09.693191; this version posted December 11, 2025. The copyright holder for this preprint (which was not certified by peer review) is the author/funder, who has granted bioRxiv a license to display the preprint in perpetuity. It is made available under a CC-BY 4.0 International license.

Supp. Fig. 4: Further control experiments and analyses for across-subjects optogenetic inhibition experiments.

(A) Blood glucose levels at baseline and during the anticipatory phase for both experimental groups in the 'light ON' experimental day. There was a significant increase in blood glucose for both groups in the anticipatory phase ($n_{tdTomato}$ = 17, $n_{ChrimsonR}$ = 11, p-value$_{tdTomato}$ = 0.0003, p-value$_{ChrimsonR}$ = 0.0004).

(B-C) No correlation between blood glucose and insulin levels during the anticipatory phase for the control cohort ('B'; n=17, R = 0.3, p = 0.25) and inhibition cohort ('C'; n=11, R = -0.13, p = 0.7), indicating that the increase in blood glucose in the optogenetic inhibition day does not explain anticipatory insulin release.

(D) Raw insulin values for all experimental groups in the across-subjects optogenetic experiment (for harvesting tissue). Control group (dTomato, n=17) showed the expected increase in both experimental days ($p_{No\ light}$= 0.00003 and $p_{Light\ on}$=0.0002). The experimental group (ChrimsonR, n=11) showed the expected increase during the 'No light' day but an attenuated response during the 'Light ON' day ($p_{No\ light}$= 0.0007 and $p_{Light\ on}$=0.002).

(E) Change in insulin levels in the across-subjects experiment between the optogenetic inhibition day and the control day for the control group (expressing tdTomato), and inhibition group (expressing ChrimsonR). There was a trend towards attenuation of anticipatory insulin release during the optogenetic inhibition day only in the ChrimsonR mice cohort (8/11 mice, p=0.12, not significant due to one subject with a very strong opposite effect, see 'F').

(F) Normalization of the data from 'E' to each mouse's baseline levels shows a significant decrease in anticipatory insulin upon InsCtx inhibition. To correct for the high variability observed in the data, we normalized it to baseline [(Anticipatory - Baseline)/Baseline]. This showed an effect of InsCtx inhibition in the ChrimsonR cohort but not in the tdTomato cohort ($n_{tdTomato}$ = 17, $n_{ChrimsonR}$ = 11, p-value = 0.037).

(G) All mice consumed the total amount of Ensure given (1 mL) in all experimental days ($n_{tdTomato}$ = 17, $n_{ChrimsonR}$ = 11).

(H) Western blot analysis of phosphorylation levels of liver Akt and GSK3b on specific phosphosites (s473 and s9, respectively) at different timepoints (Baseline, and 15, 20 and 25 minutes after start of consumption of 1 mL Ensure, lanes for each category are green (B: baseline), pink (M-O +15': meal onset +15'), yellow (M-O +20': meal onset +20') and light blue (M-O +25': meal onset +25')). The highest level of phosphorylation for both phosphosites was at 15 minutes post meal onset. We therefore chose this timepoint for tissue collection in following experiments.

(I) *Top:* Percentage of significant phosphosites that are down-regulated or up-regulated out of all significant phosphosites (FDR<15%). (Fischer's exact test. p = 3.57e-50 vs. background. *Bottom:* Percentage of phosphosites that are down-regulated or up-regulated in "background phosphosites", defined as datapoints with a log2(foldchange) between -0.5 and 0.5.

(J) Histogram of adjusted p-values for log2(fold change) of shuffled data with 1000 permutations randomly divided into two groups of n=11 and n=17 (gray) and adjusted p-values of original data (purple). Normalized frequency= counts in bin/total counts * 100. Red line: p-value=0.0541 with 5% FDR, and green line: p-value 0.13 with 15% FDR.

(K) Percentage of phosphosites related to different aspects of metabolism in the background phosphosites (datapoints with a log2(fold change) between -0.5 and 0.5). Insulin signaling (blue), Lipid metabolism (green), Carbohydrate metabolism (red) and Gluconeogenesis (yellow), light shade indicates down-

bioRxiv preprint doi: https://doi.org/10.64898/2025.12.09.693191; this version posted December 11, 2025. The copyright holder for this preprint (which was not certified by peer review) is the author/funder, who has granted bioRxiv a license to display the preprint in perpetuity. It is made available under a CC-BY 4.0 International license.

regulation and darker shade up-regulation. There was no significant bias towards either up-regulated or down-regulated phosphorylation.

Data are presented as individual measurements (points) and mean (vertical bars) ± SEM. Gray dashed lines represent the same mouse. P values were calculated by Student's paired two sample t-test (for **A**), Spearman's rank test (for **B**), Pearson R (for **C**), Wilcoxon (**E**), Mann-Whitney (**F**), Fischer's exact test (for **I,K**). *= p<0.05, ***** = p <0.00001.
